# Validation of a Classroom Version of the Eating in the Absence of Hunger Paradigm in Preschoolers

**DOI:** 10.3389/fnut.2021.787461

**Published:** 2022-01-05

**Authors:** Emily E. Hohman, Katherine M. McNitt, Sally G. Eagleton, Lori A. Francis, Kathleen L. Keller, Jennifer S. Savage

**Affiliations:** ^1^Center for Childhood Obesity Research, Pennsylvania State University, University Park, PA, United States; ^2^Department of Nutritional Sciences, Pennsylvania State University, University Park, PA, United States; ^3^Frank Porter Graham Child Development Institute, The University of North Carolina, Chapel Hill, NC, United States; ^4^Department of Biobehavioral Health, Pennsylvania State University, University Park, PA, United States

**Keywords:** eating in the absence of hunger, disinhibited eating, children, measure, eating behavior

## Abstract

Eating in the absence of hunger (EAH), a measure of children's propensity to eat beyond satiety in the presence of highly palatable food, has been associated with childhood obesity and later binge eating behavior. The EAH task is typically conducted in a research laboratory setting, which is resource intensive and lacks ecological validity. Assessing EAH in a group classroom setting is feasible and may be a more efficient alternative, but the validity of the classroom assessment against the traditional individually-administered paradigm has not been tested. The objective of this study was to compare EAH measured in a classroom setting to the one-on-one version of the paradigm in a sample of Head Start preschoolers. Children (*n* = 35) from three classrooms completed both classroom and individual EAH tasks in a random, counterbalanced order. In the group condition, children sat with peers at their classroom lunch tables; in the individual condition, children met individually with a researcher in a separate area near their classroom. In both conditions, following a meal, children were provided free access to generous portions of six snack foods (~750 kcal) and a selection of toys for 7 min. Snacks were pre- and post-weighed to calculate intake. Parents completed a survey of their child's eating behaviors, and child height and weight were measured. Paired *t*-tests and intraclass correlation coefficients were used to compare energy intake between conditions, and correlations between EAH intake and child BMI, eating behaviors, and parent feeding practices were examined to evaluate concurrent validity. Average intake was 63.0 ± 50.4 kcal in the classroom setting and 53.7 ± 44.6 in the individual setting, with no significant difference between settings. The intraclass correlation coefficient was 0.57, indicating moderate agreement between conditions. Overall, the EAH protocol appears to perform similarly in classroom and individual settings, suggesting the classroom protocol is a valid alternative. Future studies should further examine the role of age, sex, and weight status on eating behavior measurement paradigms.

## Introduction

Eating in the absence of hunger (EAH) measures the propensity to eat beyond satiety in response to the presence of highly palatable foods (1). EAH intake is a measure of hedonic eating (eating for pleasure, as opposed to homeostatic eating or in response to energy needs) (2) and is assumed to be an individual characteristic reflecting poorer appetite self-regulation. The underlying mechanisms that determine a child's tendency to engage in EAH are not well established, but may involve increased sensitivity and reactivity to food cues (i.e., “bottom-up” approach responses), and/or reduced capacity to self-regulate and inhibit such responses (i.e., “top-down” regulatory strategies) (3). Children with poorer ability to recognize internal satiety cues may also be at increased risk for EAH. EAH is associated with child overweight cross-sectionally (4–8) and has been shown to predict later adiposity (9, 10) and binge eating (11). Children who exhibit high EAH are particularly susceptible to excess energy intake in the current environment in which highly palatable foods are readily available. Exposure to food cues increases as children get older and spend more time in a variety of eating contexts. Thus, preventing increases in EAH early in life, especially among susceptible children, is an important target for obesity prevention.

The EAH task is traditionally conducted one-on-one in a controlled laboratory setting. Children consume a standardized meal to satiation followed by an *ad libitum* snack period in which they are provided with a variety of typically energy-dense, nutrient-poor foods, as well as alternative activities (e.g., toys, coloring supplies). EAH is operationalized as the number of calories consumed during the free-access period. Allowing for a high level of control and internal validity, EAH measured individually, in a laboratory or controlled environment, is considered the gold standard. Yet, assessing individual children in the laboratory is resource intensive. Not only are laboratory-based designs costly for the researcher, but requiring families to come to laboratory visits can limit the representativeness of a study sample, thus threatening external validity. Given that 61% of 3–5-year-olds in the US are enrolled in center-based childcare (12) and enrolled children eat up to 75% of their daily meals and snacks there (13), collecting EAH data in the classroom will allow researchers to access larger and more diverse samples of children in a cost-effective manner.

Although the feasibility of conducting EAH in a group classroom setting has been shown in previous studies (8, 14, 15), classroom assessment paradigms have not been validated against the traditional EAH task. Peer-influence in a group setting has the potential to change EAH kcal intake for some children. For example, data indicate children eat more snacks in the presence of friends vs. parents (16) and in larger vs. smaller groups (17). Therefore, it is critical to understand whether a classroom EAH protocol captures the same eating behavior construct as the laboratory task that was used in the majority of foundational literature on children's EAH behavior and its link with longer-term health outcomes. The purpose of this study is to compare a modified EAH protocol that can be conducted with a group of children in a classroom setting to the more traditional one-on-one version of the task in the same children. Furthermore, a limitation of earlier feasibility studies of EAH in a group setting is that known correlates of EAH and factors that have been shown to moderate the association between EAH and adiposity (i.e., child sex) were either not examined (15), or revealed mixed findings (8, 14). Therefore, this study also aims to determine concurrent and external validity by examining whether EAH is associated with child body mass index *z*-scores (BMIZ), and parent-reported child eating behaviors and whether observed associations hold across settings and among subgroups of children (e.g., age, sex, weight status).

## Materials and Methods

### Participants

Participants were child-caregiver dyads recruited from three Head Start classrooms in Central Pennsylvania in fall 2019. A study information packet, including consent forms and a caregiver survey, was sent home with all children. An opt-out process was used for children's participation; parents who did not wish their child to participate in data collection completed an opt-out form and returned the form to their child's classroom. Children with allergies to the study foods were not eligible to participate. Caregivers were also invited to complete a survey packet, which included an implied consent form stating that caregivers could consent to their own participation by completing and returning the survey to their child's classroom or by mail through a prepaid envelope. Caregivers received a $20 gift card as compensation for completing the survey. Caregiver participation in the survey was not a requirement for child participation. Of 52 packets distributed, 3 parents elected to opt their child out of the study. Of 49 enrolled children, 35 completed both the classroom and individual EAH sessions. Reasons for incomplete data include child absence on the day of the classroom EAH (*n* = 5) or child refusal of the individual EAH session (*n* = 9; no children refused participation in the classroom session). Of these 35, 33 had completed caregiver questionnaires and 30 had BMI measurements.

### Procedure

Participating children completed the EAH task in the preschool setting on two separate days under two conditions: (1) individually with a research assistant in a private space near the child's classroom (e.g., nearby office or conference room), and (2) with their classmates in a group session. Order of the two tasks was randomly assigned and counterbalanced, such that half of the children in each classroom completed the individual condition before the classroom condition, and half completed the individual condition after the classroom condition. In one classroom, 4 children who were absent on the day of the classroom EAH task completed the task together in the classroom on a separate make-up day. All procedures were approved by the Penn State University Institutional Review Board.

### EAH Tasks

The EAH task was conducted after a Head Start-provided lunch in 2 classrooms and after breakfast in the third. For both the individual and classroom conditions, research staff recorded an estimate of children's intake at the lunch or breakfast meal (i.e., ate none, less than half, more than half, or all of food initially served). Both EAH tasks occurred within 20 min of completing the meal. For the individual condition, children were invited after a meal to join a research assistant in a separate area to complete the task. Children were seated at a desk or table with the research assistant next to them. The researcher then read children a brief story to orient them to a hunger/fullness scale consisting of drawings of a child with empty, half-full, and full stomachs. Children's comprehension of the scale was assessed, and they were asked to use the scale to rate their own current feeling of hunger/fullness. Because of limitations around rescheduling the classroom condition, we did not exclude or reschedule children who reported they were hungry, but instead used hunger rating to identify a subset of children for sensitivity analysis. Children were then asked to taste and rate small pieces of 6 snack items as “Yummy”, “Just OK”, or “Yucky” using a three point, smiley face scale. Once the taste ratings were completed, children were presented with a selection of small toys and generous portions of 6 snack foods (total available kcal=758): cheese crackers (22 g), corn chips (20 g), cheese puffs (18 g), mini chocolate sandwich cookies (28 g), fruit snacks (46 g), and mini shortbread fudge cookies (28 g) (Supplemental Table 1). Researchers told the children they were to sit quietly while they did some work elsewhere in the room, and that they could eat any of the snacks and play with any of the toys while they waited for the researcher to return. Children were given 7 min of free access to the snacks and toys; researchers stood away from children but within eyeshot during the free access period, and verbally checked on the child halfway through the 7 min period (“Is everything ok? I just have a little bit more work to do—I'll come back in a few more minutes and get the foods and toys”). At the end of 7 min, the snacks and toys were removed and children returned to their classroom.

In the classroom condition, after breakfast or lunch was completed, children moved to their classroom's circle time area where one research assistant read the story explaining the hunger/fullness rating scale to the class, while other research assistants set up for the EAH task at the classroom's lunch tables. Cardboard dividers were used to designate an individual section of the table for each child. Children were dismissed from the circle-time area back to the table, where researchers asked each child to rate their current level of hunger using the picture scale. Plates with sampling portions were distributed and children were told to refrain from touching or eating the samples until instructed to do so. One research assistant led the classroom in tasting each of the six samples together, instructing children to indicate their rating of each food by holding up a picture of the corresponding “yummy,” “just ok,” and “yucky” faces. Other research assistants (1 per 3–4 children) recorded each child's responses. Once the taste test was completed, the snacks and toys (identical to those used in the individual condition) were distributed to each child. Children were instructed that they had 7 min of ‘activity time' where they could eat any of the snacks and play with any of the toys, but that they must stay in their seat, play quietly, and not take any snacks or toys from other children at the table. Research assistants monitored the children, reminded them of the rules as needed, and collected any dropped food for later weighing. For both conditions, snacks were pre- and post-weighed to determine consumption. Energy intake in kcals was determined using manufacturers' information.

### Anthropometrics

Children's height and weight were measured by trained research staff using a portable stadiometer (Model 217; Seca Corporation) and digital scale (Model 876; Seca Corporation) on a separate day. Weight was measured in duplicate to the nearest 0.1 kg, with a third measure taken if the first two differed. Height was measured in duplicate to the nearest 0.1 cm, with a third measure taken if the first two differed by more than 1 cm. Height and weight were used to calculate age- and sex-specific BMI z-scores and percentiles using the 2000 CDC Growth Charts (18). Overweight was defined as a BMI ≥85th percentile.

### Caregiver Questionnaire

Caregivers completed a survey packet that included demographic questions and measures of food security, feeding practices, and child eating behaviors. Food security was assessed using the 18-item USDA Household Food Security Module (19). Participant households were classified as food insecure if their score was 3 or greater. Children's appetitive traits were assessed using the Child Eating Behavior Questionnaire (20). We examined three subscales that were theoretically relevant to EAH—food responsiveness (α = 0.82), enjoyment of food (α = 0.89), and emotional overeating (α = 0.89). Though the satiety responsiveness scale was also potentially relevant, it had poor reliability in this sample (α = 0.40), so we did not analyze it further. Parents also completed the Eating in the Absence of Hunger Questionnaire (EAH-Q) for their child (21). The EAH-Q consists of three subscales—EAH in response to External Eating cues (α = 0.73), in response to Negative Affect (α = 0.93), and in response to Fatigue/Boredom (α = 0.89)—as well as a total score (α = 0.89).

### Statistical Analysis

Data were analyzed using SAS 9.4 (SAS Institute, Cary, NC). Associations among meal intake and hunger ratings were assessed using Spearman correlations. Differences in kcal intake between the two conditions were assessed using a paired *t*-test. Intraclass correlation coefficients were calculated using a freely available SAS macro (22), and a Bland-Altman plot was generated to examine agreement between the two protocols. We also repeated these analyses after excluding children who did not eat any of the meal served (*n* = 4) or did not indicate they were half-full or all the way full (*n* = 10) at one or both meals. To assess validity, Pearson correlations between EAH kcal in both conditions and theoretically related constructs derived from literature were examined. Theoretical correlates included child BMI *z*-score, age, total and subscale scores from the EAH-Q, and child appetitive traits from the CEBQ (23). To further explore the validity of the classroom EAH protocol, all analyses were performed in subgroups of participants divided by age, sex, and weight status. The study had a power of 0.90 to detect a 35-kcal difference in EAH kcal intake between settings, and for this primary analysis, statistical significance was considered as *p* < 0.05. The study was not powered for subgroup analyses or validity correlations with parent-reported measures, so these analyses are reported with a focus on effect sizes rather than statistical significance, and should be considered exploratory.

## Results

### Participants

Child and caregiver demographic characteristics are listed in Table 1. Children were on average 4.1 years old, non-Hispanic white (79.3%) or Hispanic/Latino (20.7%), and nearly half had BMI percentiles in the overweight or obese range. Caregivers were predominantly female parents. Consistent with Head Start eligibility, families were lower income, with approximately two-thirds participating in the Supplemental Nutrition Assistance Program (SNAP), and a quarter of households were classified as food insecure.

**Table 1:** Participant characteristics for *n* = 35 children included in analysis.

**Variable**	**Mean (SD) or *n* (%)**
**Child characteristics**	
Age, years	4.1 (0.6)
Sex, % female	16 (45.7%)
Race-ethnicity, %	
Non-Hispanic white	23 (79.3%)
Hispanic or Latino	6 (20.7%)
Overweight (BMI ≥ 85th percentile), %	14 (46.7%)
**Parent characteristics**	
Age, years	30.7 (5.9)
Sex, % female	30 (90.9%)
Race-ethnicity, %	
Non-Hispanic white	23 (79.3%)
Hispanic or Latino	6 (20.7%)
Relationship to child, %	
Parent	31 (93.9%)
Grandparent	2 (6.1%)
Highest educational level completed, %	
Less than high school	5 (15.2%)
High school graduate	22 (66.7%)
College graduate	6 (18.2%)
Relationship status, %	
Married	11 (33.3%)
Not married but living with partner	9 (27.3%)
Single	9 (27.3%)
Divorced/separated	4 (12.1%)
Income, %	
< $20,000	7 (21.2%)
$20,000–49,999	12 (36.4%)
≥$50,000	7 (21.2%)
Do not know	11 (33.3%)
Employment, %	
Employed full time	13 (39.4%)
Employed part-time	10 (30.3%)
Student	1 (3.0%)
Unemployed	7 (21.2%)
Other	2 (6.1%)
Overweight/obesity, %	21 (72.4%)
Supplemental Nutrition Assistance Program (SNAP) participant, %	21 (63.6%)
Special Supplemental Nutrition Program for Women, Infants, and Children (WIC) participant, %	20 (62.5%)
Household food insecurity, %	8 (24.2%)

*Sample size varies for each variable due to missing data*.

### Meal Intake and Hunger Rating

Prior to the classroom EAH session, 6% of children consumed all of what they were initially served at their Head Start-provided meal, 60% consumed more than half, 26% consumed less than half, 6% consumed nothing, and 1 child (3%) had missing data due to observer error. In the hunger assessment, 57% selected the “full” stomach image, 20% selected “half-full,” 17% selected “empty,” and 6% did not select an answer. Prior to the individual EAH session, 20% consumed all of their meal, 49% consumed more than half, 29% consumed less than half, and 3% ate nothing. In the hunger assessment, 63% selected the “full” stomach, 14% selected “half-full,” 9% selected “empty,” and 14% did not provide an answer. There were no significant associations between amount consumed at the meal and hunger rating, nor any associations between these variables and amount consumed in the EAH task in either setting. Hunger ratings in the individual and classroom settings were significantly correlated (ρ = 0.53, *p* = 0.003).

### Liking of Test Foods

All six foods provided were generally liked by the children and ratings were similar between the two conditions. During the classroom condition, 73–80% of children rated each food as “yummy” with 2–11% rating them as “just ok”; in the individual condition 69–81% rated the foods as “yummy” and 6–16% rating them as “just ok”. Liking did not significantly predict intake.

### Classroom vs. Individual EAH

**Figure 1:**
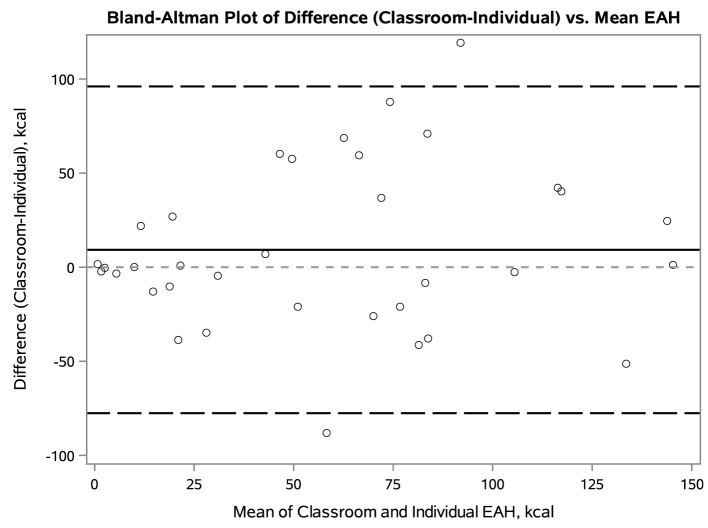
Bland-Altman Plot for total kcal intake in classroom and individual Eating in the Absence of Hunger paradigms in preschoolers. The solid line represents the mean difference of classroom minus individual kcal intake. The dark dashed lines represent 2 standard deviations from the mean.

Total intake, sweet and salty subcategories, and individual foods, are listed in Table 2. Children consumed an average of 63.0 ± 50.4 kcal in the classroom condition and 53.7 ± 44.6 kcal in the individual condition. The mean difference between settings was 9.2 ± 43.4 kcal; this difference was not statistically significant in a paired *t*-test (*p* = 0.22). The intraclass correlation coefficient for kcal intake was 0.57 (95% confidence interval: 0.35–0.77), indicating moderate agreement. Examination of a Bland-Altman plot (Figure 1) did not indicate systematic differences in agreement by magnitude of EAH. A sensitivity analysis restricting the sample to children who ate something at the breakfast/lunch meal and indicated they were half full or completely full prior to both EAH sessions (*n* = 21) produced similar results. The mean difference in the paired *t*-test was 14.2 ± 47.3 kcal (*p* = 0.18) and the intraclass correlation coefficient was 0.55 (95% confidence interval: 0.26–0.80).

**Table 2:** Kcal intake in classroom vs. individual eating in the absence of hunger (EAH) tasks (*n* = 35 children).

**Item**	**Kcal served**	**Classroom setting, kcal consumed**		**Individual setting, kcal consumed**		**Paired *t*-test**	**Intraclass correlation (ICC)**	
		**Mean (SD)**	**Range**	**Mean (SD)**	**Range**	***P*-value**	**ICC**	**95% CI**
Cheese cracker	111	3.3 (5.7)	0–18.5	4.0 (5.1)	0–18.0	0.49	0.43	0.20–0.70
Corn chip	114	4.6 (6.1)	0–28.6	2.4 (2.8)	0–10.3	0.04	0.18	0.02–0.66
Cheese puff	103	5.7 (11.8)	0–52.5	4.3 (6.2)	0–20.0	0.46	0.31	0.10–0.65
Chocolate sandwich cookie	130	13.7 (22.5)	0–76.8	9.2 (22.0)	0–84.5	0.22	0.51	0.28–0.74
Fruit snacks	160	21.2 (30.9)	0–134.3	23.9 (31.1)	0–94.7	0.50	0.71	0.53–0.85
Shortbread fudge cookie	140	14.4 (21.4)	0–87.0	9.9 (15.0)	0–56.5	0.13	0.54	0.31–0.75
Total salty	328	13.6 (17.3)	0–63.8	10.7 (10.0)	0–34.6	0.32	0.28	0.08–0.64
Total sweet	430	49.4 (47.0)	0–143.1	43.0 (42.9)	0–142.0	0.32	0.65	0.44–0.81
Total	758	63.0 (50.4)	0.5–156.0	53.7 (44.6)	0–159.2	0.22	0.57	0.35–0.77

For sweet and salty subcategories as well as individual foods, there were no differences in intake between the two conditions, except for corn chips; more corn chips were consumed in the classroom than in the individual condition (4.6 ± 6.1 vs. 2.4 ± 2.8 kcal, *p* = 0.04). Intake of, and agreement between settings, was generally higher among sweet foods than salty items. *T*-tests and ICCs for sweet and salty subcategories and individual foods in the sample restricted by meal intake/fullness rating were similar to those in the full sample.

### Validity Analysis

Correlations between EAH kcal in both conditions and theoretically related constructs are provided in Table 3. There were no statistically significant correlations between EAH in either condition with child BMI *z*-score or parent report of child appetitive traits from the CEBQ. There were marginally statistically significant correlations between parent-reported EAH (total and negative affect and external eating subscales) and individual EAH kcal intake (Table 3), but not with classroom EAH kcal. BMI-z and parent reports of child eating behavior were not correlated with difference in kcal intake between the two conditions. In the subset restricted by meal intake/fullness rating, stronger correlations between parent-reported variables and EAH intake were observed, particularly for the classroom condition (Supplemental Table 2).

**Table 3:** Correlations between eating in the absence of hunger (EAH) kcal in the classroom and individual settings, and theoretically related constructs.

		**Correlations**					
		**Classroom EAH kcal**		**Individual EAH kcal**		**Difference (Classroom-individual)**	
**Variable**	**Mean (SD)**	***r***	***p***	***r***	***p***	***r***	***p***
BMI *z*-score (*n =* 30)	1.08 (1.03)	−0.11	0.57	−0.004	0.98	−0.11	0.56
**Parent report of child appetitive traits (child eating behavior questionnaire) (*****n** **=*** **33)**							
Food responsiveness	2.1 (0.7)	0.05	0.79	−0.09	0.62	0.14	0.42
Enjoyment of food	3.4 (0.7)	−0.11	0.54	−0.10	0.59	−0.03	0.88
Emotional overeating	1.4 (0.6)	0.19	0.28	0.10	0.56	0.11	0.55
**Parent report of child eating in the absence of hunger (EAH-Q) (n** **=** **33)**							
Total score	1.8 (0.6)	0.19	0.28	0.33	0.06	−0.12	0.52
Negative affect	1.2 (0.5)	0.18	0.33	0.31	0.08	−0.12	0.51
External eating	2.9 (0.8)	0.13	0.45	0.33	0.06	−0.18	0.31
Boredom	1.5 (0.8)	0.19	0.29	0.20	0.27	0.01	0.95

*Both questionnaires have answers ranging from 1 (never) to 5 (always)*.

### Subgroup Analyses

#### Age

Age subgroups included 3 year-olds (*n* = 13) and 4–5 year-olds (*n* = 19). Among 3 year-olds, there was no difference in kcal intake between the classroom and individual conditions (56.3 ± 40.2 vs. 58.5 ± 48.5, *p* = 0.79). The ICC for 3 year-olds was 0.81 (95% CI: 0.49–0.95), indicating good agreement. In contrast, 4–5 year olds tended to consume more calories in the classroom setting than the individual setting (69.5 ± 54.6 vs. 50.6 ± 44.9, *p* = 0.12). The ICC for 4–5 year olds was comparatively poorer than 3 year-olds at 0.45 (95% CI: 0.17–0.76). There was no significant difference by child age group in kcal consumed for either condition.

#### Sex

Girls (*n* = 16) tended to consume more calories in the classroom relative to the individual setting (62.2 ± 43.7 vs. 44.8 ± 36.8, *p* = 0.051). In contrast, boys (*n* = 19) consumed similar amounts in both settings (63.6 ± 56.6 vs. 61.3 ± 50.1, *p* = 0.84). The ICCs for girls (0.59, 95% CI: 0.28–0.84) and boys (0.55, 95% CI: 0.26–0.81) were similar to each other and to the sample overall. There was no significant difference by child sex in kcal consumed for either condition.

#### BMI

Children with overweight (*n* = 14) consumed similar amounts in both the classroom and individual conditions (54.0 ± 39.1 vs. 49.7 ± 44.9, *p* = 0.84), and had an ICC of 0.60 (95% CI: 0.27–0.86), indicating moderate agreement between conditions. Children with normal weight (*n* = 16) tended to consume more in the classroom than in the individual session (71.7 ± 54.2 vs. 55.2 ± 49.4, *p* = 0.21) and had a lower ICC at 0.49 (95% CI: 0.18–0.81). There was no significant difference by weight status in kcal consumed for either condition.

## Discussion

This study examined the validity of the EAH paradigm in a classroom (i.e., group) setting compared to the classic individual setting and found no significant difference between classroom and individual EAH total kcal intake in preschoolers aged 3–5 years. When examined by age, sex, and weight status, the two protocols performed most similarly for younger children, boys, and children with overweight, while older preschoolers, girls, and normal weight children tended to consume more calories in the classroom compared to the individual condition. However, the mean differences between conditions in these groups were small (<20 kcal) and do not suggest reduced validity of the paradigm in these subgroups. This is the first study to test the validity of a preschool classroom-based EAH protocol in comparison with a more traditional, one-on-one version of the paradigm.

In this within-subjects design, total kcal intake did not differ significantly between the two conditions, and examination of the Bland-Altman plot did not reveal any systematic differences by average kcal intake between classroom and individual EAH. This suggests that the EAH classroom protocol is a valid alternative to the well-established individual paradigm. Results were similar after excluding children who did not eat at the meal prior to EAH or did not report feeling at least half full in one or both conditions. Average total EAH kcal intake in this study was 53.7 kcal and 63.0 kcal (7–8% of offered kcal) in the individual and classroom settings, respectively. This is similar to other reports in preschool aged children, where average intake ranged from 55 to 90 kcal in classroom-based protocols (14, 15) and 36–216 kcal in laboratory studies (1, 9, 24).

Though we used cardboard dividers to provide some separation and instructed children not to talk to their classmates, with frequent reminders during the task, these protocols were not entirely effective at preventing children from interacting with or looking at their peers during the task. Potential effects of this social setting were seen in older children, who consumed more kcals in the classroom setting than in the individual setting. This is consistent with previous literature showing that preschoolers ate more of a snack when seated in larger vs. smaller groups (17). Additionally, it has been shown that children aged 5–7 years consumed more unhealthy snacks in the presence of friends than in the presence of their mother, but healthy snack consumption did not differ (16, 25), while children 5–11 years ate more cookies with their sibling than with an unfamiliar peer or when alone (26). These data suggest that, even with dividers to block children's view of each other, classroom EAH kcal intake may be subject to peer influence, especially among older children. However, this may in fact increase ecological validity of the EAH task as overeating commonly occurs in social settings. Conducting the EAH task in the classroom group setting may also tap aspects of general self-regulation in addition to appetite self-regulation. In the classroom protocol, children were required to wait for others to be served before eating the snack foods and to refrain from interacting with their peers. In the one-on-one condition, research assistants were able to exert more direct control over the protocol. Future studies using the classroom protocol should consider including a measure of general self-regulation to use as a covariate.

In contrast to previous findings, our data show that girls and children with normal weight consumed more kcals in the classroom setting than in the individual setting, though most of this previous work was with older children. Salvy and colleagues reported that adolescent girls (but not boys) and children with overweight (but not normal weight) ate more healthy and fewer unhealthy snacks in the presence of friends than in the presence of their mother (16, 25). Among 7–9 year olds completing EAH in a classroom setting, there was a linear association between weight status and EAH for boys, but a quadratic relationship in girls, such that girls with overweight and obesity had slightly lower EAH than girls with BMIs between 50 and 85th percentile. The authors suggested that this may reflect overweight girls responding to the task in a way they consider to be socially desirable, such as limiting intake (8). In primary school children, the effect of peers' eating on participant snack intake differed by participant weight status. Children with overweight ate more when a peer ate a large portion of the snack, while children with normal weight ate less when the peer did not eat the snack (27). Taken together, these findings suggest that girls and children with overweight are more likely to limit their intake in a group setting based on social norms. However, in our preschool age sample, girls and children with normal weight, but not overweight, consumed more in the classroom vs. individual setting. Further work is needed to clarify how the role of peer influence on eating evolves through development, particularly for girls and children with overweight.

Contrary to our expectations, we did not find significant associations between EAH in either setting and theoretically-related parent-reported appetitive behaviors from the CEBQ. It is likely that our small sample size increased the risk for type II error. Additionally, appetitive traits and EAH were measured in different settings (at home as reported by parents and observed in preschool, respectively), so it is possible that the correlations would be stronger if a teacher reported child appetitive traits. The CEBQ also asks questions about eating behaviors in general, while the EAH tasks taps behavior in a very specific situation, i.e., free access to palatable snacks following a meal. However, other studies examining the relationship between EAH kcal and such variables in this age group have also had mixed findings. For example, food responsiveness was not significantly correlated with EAH in our sample. Other groups have found a small positive (*r* = 0.19) (28) or no correlation (29–32) between food responsiveness and EAH. Enjoyment of food was not correlated with EAH, which is consistent with other studies (28, 30). For emotional overeating, we observed a positive but not statistically significant association with EAH kcal in both settings; similar findings have been previously reported (*r* = 0.13–0.15) (28, 29). We did observe correlations around *r* = 0.3 between parent-reported child EAH and EAH kcal in the individual condition, which approached statistical significance, but smaller correlations with EAH kcal in the classroom condition. In the restricted subsample of children who ate at the meal and reported fullness before both EAH conditions, correlations with the EAH-Q were higher and more similar between the two conditions. The EAH-Q has been primarily used in older children (21, 33, 34), but our results suggest that this questionnaire is predictive of observed EAH behavior in preschoolers, particularly if the observations are made truly “in the absence of hunger”; however, confirmation in larger samples is required.

We found no significant association between BMI-z and total EAH kcal in either setting. While a systematic review found that EAH was positively associated in both cross-sectional and longitudinal studies with child weight status among children 12 years of age or younger (23), the evidence for this relationship specifically in preschool age children is somewhat more mixed. In Fisher and Birch's original study of 3–5 year olds, weight-for-height was positively associated with child kcal intake for girls (*r* = 0.38) but not boys (*r* = −0.08) (1). Kcal consumed in an EAH session was positively associated with BMI-z in French preschoolers (*r* = 0.14) (14) and U.S. Hispanic children in Head Start (*r* = 0.20) (31), while other studies have found no association between EAH and BMI or weight status in young children (30, 32, 35, 36). Our ability to detect an association between BMI an EAH may have been limited due to the small sample size and relatively high average BMI among this sample; nearly half of children had a BMI exceeding the 85th percentile. Variation in EAH protocols (e.g., standardization of meal, length of delay between meal and free access, foods offered, length of free access period) also makes comparisons between studies challenging. Future research should determine optimal configurations of the EAH task to best detect a phenotype that is associated with adverse health outcomes.

Most EAH kcal intake in this study came from sweet foods. Additionally, we found that intake of certain salty foods differed between classroom and individual EAH (i.e., children ate significantly more corn chips in the classroom). Overall intake of sweet foods was more similar between the two settings than salty foods. Revisions to salty snack offerings (e.g., substituting another sweet for one of the salty snacks) could be considered in future studies as it may improve agreement. Anecdotal feedback from our research team suggested that some of the toys that were included in our protocol (i.e., magnetic building pieces) were particularly novel and exciting for the children and may have been more rewarding than the foods offered. Including toys that provide an alternative activity to eating, but that are not overly interesting may improve the concurrent validity (e.g., EAH association with BMI-z) in future research.

Findings from this study should be interpreted in consideration of the study's strengths and limitations. Strengths include a randomized, counter-balanced crossover design and using objective measures of height and weight to calculate BMI. We also made improvements to our protocols [e.g., use of individual (rather than shared toys) for each child and teaching children about the concept of hunger prior to the EAH task (with a story book)], based on recommendations from a prior study that examined feasibility of classroom EAH (15). This study also had limitations. We used school-provided meals, which, while cost saving, gave us little control over what was offered. We conducted the individual EAH sessions in areas outside the classroom but still within Head Start facilities; while this protocol replicates the one-on-one nature of the classic laboratory EAH paradigm, it may still be a more familiar environment to children than a research laboratory. Additionally, while sufficiently powered to detect a meaningful difference in calorie intake between the two conditions, the sample size was relatively small, which may preclude our ability to detect associations between EAH and other measures.

In conclusion, findings from this study provide preliminary support for the validity of a group-based EAH protocol in preschool classrooms compared to the classic individual task. Standardization of the meal and other measures to ensure children are not hungry prior to the free access period may help to improve validity. Further testing in larger samples may help to confirm validity of the EAH classroom task and allow for better exploration of the differences in appetite self-regulation across age, sex, and weight status in eating behavior studies. In conclusion, a classroom-based EAH task will allow for broader application of assessing EAH in studies with young children.

## Data Availability Statement

The raw data supporting the conclusions of this article will be made available by the authors, without undue reservation.

## Ethics Statement

The studies involving human participants were reviewed and approved by Pennsylvania State University Institutional Review Board. Written informed consent for participation was not provided by the participants' legal guardians/next of kin because: Parents provided informed consent but written documentation requirement was waived by the Institutional Review Board. Parents were provided with study information and completed a form if they wished to opt their child out of participation of the study being conducted in their classroom.

## Author Contributions

EH, KM, SE, and JS designed and executed the study. EH analyzed the data. EH, KM, and SE drafted the initial manuscript. EH, KM, SE, LF, KK, and JS interpreted the data and critically revised the manuscript. All authors contributed to the article and approved the submitted version.

## Funding

This work was funded by NIH NIDDK grant R01DK120754. SE is funded through a postdoctoral fellowship (T32-HD07376) through Frank Porter Graham Child Development Institute, University of North Carolina at Chapel Hill.

## Conflict of Interest

The authors declare that the research was conducted in the absence of any commercial or financial relationships that could be construed as a potential conflict of interest.

## Publisher's Note

All claims expressed in this article are solely those of the authors and do not necessarily represent those of their affiliated organizations, or those of the publisher, the editors and the reviewers. Any product that may be evaluated in this article, or claim that may be made by its manufacturer, is not guaranteed or endorsed by the publisher.
