# Supplementary material for: Validation of a Classroom Version of the Eating in the Absence of Hunger Paradigm in Preschoolers
Source: Front Nutr. 2022 Jan 5;8:787461. doi: 10.3389/fnut.2021.787461 (PMC8768940; doi:10.3389/fnut.2021.787461)
Supplement: Supplementary file 1 [file Table_1.DOCX]

Supplemental Table 1: EAH foods and toys offered

| **Food** | **Brand/**  **Manufacturer** | **Amount offered (g)** | **Energy density (kcal/g)** |
| --- | --- | --- | --- |
| Mini Oreos | Nabicso | 28 | 4.8 |
| Mini Fudge-Stripe Cookies | Keebler | 28 | 5.0 |
| Fruit Flavored Snacks, assorted flavor | Mott’s | 46 | 3.5 |
| Cheez-It Orginal | Kellogg | 22 | 5.0 |
| Fritos | Frito-Lay | 20 | 5.7 |
| Cheetos | Frito-Lay | 18 | 5.7 |
|  | | | |
| **Toy** | | **Brand/Manufacturer** | |
| Magnetic building blocks | | Mibote | |
| Plastic figurines | | My Little Pony  Trolls | |
| Toy cars | | Hotwheels | |
| Crayons | | Crayola | |
| Coloring pages | | Crayola | |

Supplemental Table 2: Correlations between EAH kcal in the classroom and individual settings, and theoretically related constructs in restricted subsample (n=20)

| Variable | Classroom EAH kcal | | Individual EAH kcal | | Difference (Classroom-individual) | |
| --- | --- | --- | --- | --- | --- | --- |
|  | r | p | r | p | r | p |
| BMI z-score (n=20) | -0.27 | 0.25 | -0.10 | 0.67 | -0.18 | 0.44 |
| Parent report of child appetite (n=20) | | | | | | |
| Food Reponsiveness | 0.34 | 0.14 | 0.06 | 0.81 | 0.31 | 0.19 |
| Enjoyment of Food | 0.06 | 0.81 | 0.03 | 0.89 | 0.02 | 0.92 |
| Emotional Overeating | 0.44 | 0.052 | 0.19 | 0.41 | 0.27 | 0.25 |
| Parent report of child eating in the absence of hunger | | | | | | |
| Total Score | 0.36 | 0.12 | 0.42 | 0.07 | -0.06 | 0.79 |
| Negative Affect | 0.28 | 0.27 | 0.35 | 0.13 | -0.07 | 0.77 |
| External Eating | 0.27 | 0.26 | 0.43 | 0.06 | -0.17 | 0.46 |
| Boredom | 0.40 | 0.08 | 0.29 | 0.22 | 0.12 | 0.60 |
